# Transglutaminase-Induced Free-Fat Yogurt Gels Supplemented with Tarragon Essential Oil-Loaded Nanoemulsions: Development, Optimization, Characterization, Bioactivity, and Storability

**DOI:** 10.3390/gels8090551

**Published:** 2022-08-30

**Authors:** Seyed Mohammad Taghi Gharibzahedi, Zeynep Altintas

**Affiliations:** 1 Institute of Chemistry, Faculty of Natural Sciences and Maths, Technical University of Berlin, Straße des 17. Juni 124, 10623 Berlin, Germany; 2 Institute of Materials Science, Faculty of Engineering, Kiel University, 24143 Kiel, Germany

**Keywords:** yogurt gel, tarragon oil, transglutaminase, ultrasound-assisted emulsification, nanoemulsion, rheology, microstructure, sensory, antioxidant, antimicrobial

## Abstract

There is a high demand for designing healthy-functional dairy gels with a newly structured protein network in the food industry. Non-fat yogurt gels enriched with stable tarragon essential oil-nanoemulsions (TEO-NEs) using crosslinking of microbial transglutaminase (MTGase) were developed. The gas chromatography-mass spectrometry analysis showed that methyl chavicol (85.66%) was the major component in TEO extracted by the hydrodistillation process. The storage-dependent droplet size and physicochemical stability data of samples at room temperature for 30 days revealed that the TEO-NE containing 0.5% tween-80 and 1:2 TEO/sunflower oil had the lowest peroxide value and droplet growth ratio. The response surface methodology-based formulation optimization of free-fat yogurt gels using MTGase (0.15–0.85 U/g) and the best TEO-NE (0.5–3.02%) using the fitted second-order polynomial models proved that the combination of 0.87% TEO-NE and 0.70 U/g MTGase led to the desired pH (4.569) and acidity (88.3% lactic acid), minimum syneresis (27.03 mL/100 g), and maximum viscosity (6.93 Pa s) and firmness (0.207 N) responses. Scanning electron microscopy images visualized that the MTGase-induced crosslinks improved the gel structure to increase the firmness and viscosity with a reduction in the syneresis rate. The optimal yogurt gel as a nutritious diet not only provided the highest organoleptic scores but also maintained its storage-related quality with the lowest mold/yeast growth and free-radical oxidation changes.

## 1. Introduction

Yogurt is one of the most popular dairy products in the world, not only for its high accessibility and acceptability across different communities but also for its nutritional values and health benefits. It is manufactured by the acid gelation of milk and the conversion of lactose to lactic acid (LA) through the activity of starter cultures of *Lactobacillus delbrueckii* subsp. *bulgaricus* and *Streptococcus thermophilus*. In general, there are two basic yogurts of set-type and stirred-type [1]. In the set-type yogurts, the gel structure is allowed to form in containers during two steps of fermentation and incubation, whereas in the stirred-type, the gel structure formed within incubated large fermentation vessels is stirred during cooling to obtain a smooth and semi-viscous product before filling and packaging [1,2].

This fermented product is rich in group B vitamins, minerals (e.g., calcium, potassium, etc.), and high-quality proteins [1,3]. The consumption of yogurt products has remarkable health outcomes such as improvement of immune function; serum cholesterol and blood pressure reduction; control of gastrointestinal disorders like microbial infections, irritable bowel symptoms, diarrhea, and constipation; prevention of cardiovascular diseases and cancer types (e.g., colorectal, breast, prostate, etc.); enhancement of lactose digestion by decreasing symptoms of intolerance in lactose maldigesters; promotion of muscle growth and bone health; and reduction of diabetes risk and metabolic syndrome [4,5]. The intake of this healthy-functional product by increasing the bioavailability and accessibility of nutrients can meaningfully promote antioxidative, antimicrobial, anticarcinogenic, and hypoallergenic effects [4]. Besides this, there has been a growing demand for reduced-fat or free-fat yogurts in the market due to the role of fat in enhancing the risk of many serious diseases and disorders such as coronary heart disease, cancer, obesity, arteriosclerosis, etc. [6]. Nonetheless, this critical component plays a pivotal function in improving textural and rheological properties, organoleptic characteristics, and consumer preferences. As a result, the whole and partial replacement of fat globules of yogurt not only increase the serum syneresis by forming a weak gel structure but also can be associated with unpleasant flavor and aroma as well as sourness [7]. Hence, it is necessary to find alternative ingredients at optimal concentrations to maintain the gel strength and improve the sensory attributes of final yogurt products.

Microbial transglutaminase (MTGase), due to crosslinking reactions with milk proteins, has been used to develop low-fat dairy products (e.g., yogurt, cheese, and ice cream) with improved physicochemical and textural quality properties without any negative effect on their sensory properties [3,8]. This enzyme is known as glutaminyl-peptide-amine γ-glutamyl transferase and is commercially synthesized by *Streptomyces mobarensis*. MTGase forms covalent crosslinks of inter- or intra-molecular ε-(γ-glutamine)-lysine isopeptidic bonds through the catalysis of an acyl transfer reaction between a γ-carboxyamide group in protein-bound glutamine residues (acyl donor) and an ε-amino group in a protein-bound lysine residue (acyl acceptor) [9,10]. Hence, this enzyme with glutamine-lysine crosslinks contributes to developing strong protein networks with high molecular weights to improve the physicochemical, rheological, and textural properties of protein-based food products [10].

Nanoemulsion (NE) technology is one of the most important encapsulation strategies for water-insoluble nutraceutical ingredients such as omega-3 fatty acids and essential oils (EOs). Nanoemulsification can effectively encapsulate hydrophobic bioactive constituents into nano-scale droplets to increase the solubility in the aqueous phase, protect them against any deterioration or degradation reactions, and improve their absorption and bioavailability rate [4,11]. Currently, some studies have been performed to fortify the yogurt matrix using EO-in-water NEs. Salama et al. developed a stirred probiotic yogurt by supplementing spearmint, lemongrass, clove, and cinnamon EOs-loaded NEs [12]. Faraji et al. manufactured a reduced-fat probiotic yogurt enriched by shallot EO- NE containing omega-3 fatty acids. They reported that the presence of 1.4% nanoemulsion in the yogurt formulation due to the availability of nutrients could increase the survivability of probiotic bacteria and sensory attributes as a result of the volatile compounds released during the fermentation [13]. In recent years, the bioactivity of tarragon (*Artemisia dracunculus* L.)-EO (TEO) in terms of antioxidant, antibacterial, antimalarial, and antiinflammation has been explored due to the total phenolic content and notable quantity of methyl chavicol [14,15]. The antimicrobial/antioxidant potential of TEO to produce bioactive edible films and coatings for extending the shelf life of pork slices [16], beef slices [17], beef burgers [18], kumquat fruit [19], and brook trout (*Salvelinus fontinalis*) [20] was successfully investigated. Further, the effective role of EO of this perennial herb of the family Asteraceae has been recently demonstrated to control spoilage, improve organoleptic characteristics, and extend the shelf life of commercial and traditional yogurts in Iran [21,22]. However, no study on the use of TEO-loaded NEs to enrich yogurt products has not been yet reported.

Therefore, this study aimed at achieving the following outcomes: (i) the design and development of a stable TEO-NE using the ultrasound-assisted nanoemulsification; (ii) the formulation optimization of yogurt with MTGase and TEO-NE to achieve the best physicochemical, textural, and rheological properties; and (iii) the shelf-life assessment of the optimal yogurt in terms of antioxidant, antimicrobial, and sensory characteristics.

## 2. Results and Discussion

### 2.1. Identification and Quantification of Chemical Compounds of TEO

Table 1 shows chemical compounds and structures, Kovats indices, as well as retention times of TEO extracted by the Clevenger. The gas chromatography–mass spectrometry (GC-MS) analysis could determine 97.41% of the total composition of this EO. The most dominant constituent present in TEO was estragole or methyl chavicol (85.66%). There were some monoterpene hydrocarbons such as z-β-ocimene (4.79%), trans-β-ocimene (4.02%), limonene (0.91%), α-pinene (0.67%), β-pinene (0.19%), and β-myrcene (0.15%). Other identified chemicals were oxygenated components of (E)-isosafrole (0.45%), geranial (0.26%), 4-methoxy cinnamaldehyde (0.16%), and eugenol (0.15%). The presence of methyl chavicol as the major volatile constituent (81.89–84.83%) in TEO was earlier reported in the literature [19,23,24].

### 2.2. Physicochemical Stability of TEO-Nanoemulsions

**Figure 1:**
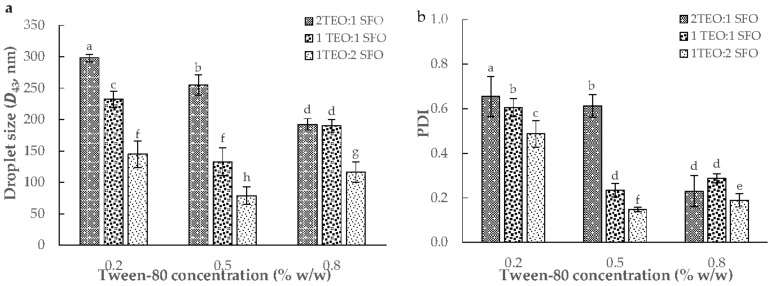
The volume-weighted mean diameter (*D*_43_, (**a**)) and polydispersity index (*PDI*, (**b**)) of TEO-NEs as a function of TEO/SFO ratio and surfactant concentration. (a–h) indicate the significant statistical difference (*p* < 0.05).

**Figure 2:**
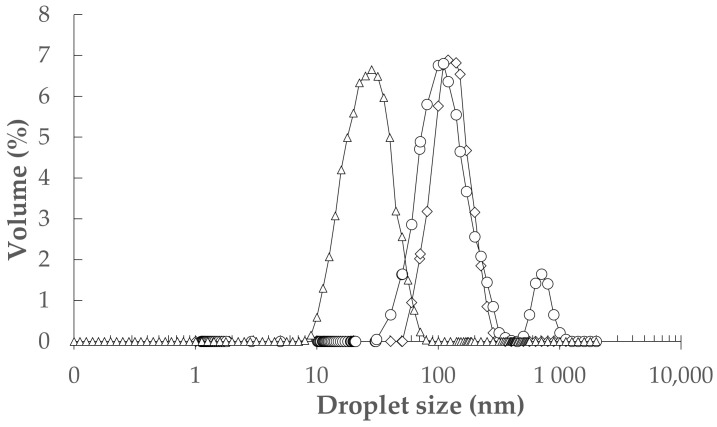
The droplet size distribution of TEO-NEs with a TEO/SFO of 1:2 at tween-80 concentrations of 0.2% (o), (∆) 0.5%, and (◊) 0.8% (*w*/*w*).

The ultrasound-assisted emulsification (UAE) was used to fabricate nine of 10% oil-in-water NEs with different TEO/sunflower oil (SFO) (2:1, 1:1, and 1:2 *w*/*w*) and tween-80 (0.2, 0.5, and 0.8% *w*/*w*). The analysis of variance (ANOVA) showed that the effect of TEO/SFO was stronger than tween-80 on the particle size and polydispersity index (*PDI*) results (*p* < 0.05). In general, an increase in the concentration of tween-80 from 0.2 to 0.8% resulted in a decrease in the droplet size and especially *PDI*. Moreover, an increase in the weight ratio of SFO to TEO diminished the volume-weighted mean diameter (*D*_43_) and *PDI* parameters (Figure 1). A formulation with 0.5% tween-80 and a TEO/SFO ratio of 1:2 led to the lowest droplet size and *PDI*, whereas the maximum *D*_43_ and *PDI* values were measured in NEs formulated with 0.2% tween-80 and TEO/SFO of 2:1 (*p* < 0.05; Figure 1). Figure 2 depicted the droplet size distribution of TEO-NEs prepared with the optimal TEO/SFO ratio of 1:2 at various tween-80 levels. NEs fabricated at the lowest tween-80 concentration were unstable and exhibited a bimodal distribution of droplet size, while those formulated with 0.5 and 0.8% tween-80 revealed a monomodal and narrower droplet size distribution (Figure 2). Improving the physical stability with an increase in the concentration of tween-80 may be attributed to the increased surface activity and the reduced interfacial tension which can effectively accelerate the diffusion rate of this small-size nonionic surfactant from the organic phase to the aqueous phase for easier adsorption onto the surface of the formed droplets. Hence, it is expectable to have stable TEO-NEs with fine droplets and narrow size distribution at a high tween-80 content because it facilitates droplet disruption during homogenization to form a stable protective layer around oil droplets for preventing emulsion destabilization [25]. Nonetheless, the decreased physical stability at 0.8% tween-80 can be due to the increase in the interfacial rheology of emulsion droplets.

Accordingly, this phenomenon can meaningfully hinder the breakup of emulsion droplets during homogenization, leading to larger particle sizes [26]. It was earlier demonstrated that excessive emulsifiers increased the self-aggregation, particle size, and viscosity of emulsion systems [27,28]. Water-insoluble SFO plays as a carrier for water-soluble TEO. The presence of TEO results in one of the most main instability mechanisms for NE systems, namely ‘Ostwald ripening (OR)’. Under this condition, this water-soluble component will diffuse from the small-size to large-size droplets and thus a higher portion of NEO molecules can be observed in the larger droplets than in the smaller ones. Owing to the entropy of mixing, structural changes and droplet size distribution of NEs will be thermodynamically undesirable. Thus, it seems that the optimal TEO/SFO ratio of the two components within the oil droplets with satisfactory solubility is 1:2, preventing OR with a diminution in the droplet growth ratio (*DGR*), as well as size distribution of droplets [29,30].

Table 2 exhibits the physicochemical stability of different NEs during storage for 30 days at ambient temperature. An increase in the storage time significantly increased the *DGR* and peroxide value (PV) levels of TEO-NEs (*p* < 0.01). Although high concentrations of tween-80 (0.5 and 0.8% *w*/*w*) led to a significant reduction in the *DGR* (*p* < 0.05), a more physical instability in NEs emulsified with 0.8% tween-80 was observed in TEO/SFO ratios of 1:1 and 1:2 (*w*/*w*) (Table 2). The lowest *DGR* was for NEs produced with 0.5% tween-80 and 1:2 TEO/SFO, particularly in the first week of storage. Increasing the surfactant concentration increased the chemical stability rate (*p* < 0.05). However, there was no significant difference in the PV between NE samples formulated with 0.5 and 0.8% tween-80 (Table 2). Generally, the best NE formulation contained 0.5% tween-80 and 1:2 TEO/SFO and chose to enrich non-fat yogurts in the next step of this study. As a result, the production of NEs with the minimum level of surfactant would be very economical from an industrial viewpoint.

The storage-dependent *DGR* confirms that the movement increase of the dispersed droplets into the continuous phase during the storage escalates the collisions and growth rate of droplets. Consequently, the coalescence of densely packed oil droplets by prolonging the storage time is the main reason for the increased droplet size and *PDI* [31]. On the other hand, the presence of a high number of fine droplets in NE systems in two initial weeks with the increased resistance to flow can effectively augment the apparent viscosity to increase the physical stability rate [32]. The oxidation instability is because of the generation of lipid hydroperoxides at the surface of droplets and also the transition of metals such as ferric ions from the aqueous phase [33]. In general, there is a high oxidation rate in the UAE process due to the high temperature and dissolved oxygen in the interface as a result of the cavitation phenomenon and acoustic waves. In the surfactant poor regime, the coalescence rate of droplets was significant because there was insufficient surfactant to fully stabilize the newly created interface. Thus, not enough tween-80 to cover newly formed smaller droplets increased their susceptibility against high temperatures and oxygen induced by the cavitation [31,32,33]. On the other hand, the reduced size of droplets with a wider specific surface area at high concentrations of tween-80 can highly provide the accessibility of prooxidants in the aqueous phase to oxidative degrade the lipid ingredients [34]. However, the favorable oxidative stability at 0.5 and 0.8% tween-80 can be attributed to the presence of α-tocopherol, which can extend the lag phase in the induction period of lipid autoxidation. Earlier, Sahafi et al. [35] and Liu et al. [36] reported that this vitamin could effectively retard the lipid autoxidation process to form primary and secondary products in walnut oil-based emulsions and NEs.

### 2.3. Optimization and Characterization of Non-Fat Yogurt Gels

#### 2.3.1. Model Fitting

Table 3 shows the levels of critical structural components and experimental responses for the optimization procedure based on a 5-level-2-factor response surface methodology-central composite rotatable design (RSM-CCRD). The multiple linear regression analysis showed that second-order polynomial models were adequately accurate to predict the relevant responses because not only were highly significant (*p* < 0.0001), but also had insignificant lack-of-fit values (Table 4 and Table 5). Table 4 reveals that the assessed regression coefficients of the fitted models for each response variable, accompanied by the corresponding coefficient of determination (*R*^2^), adjusted *R*^2^ (adj-*R*^2^), coefficient of variation (*CV*), root mean square error of prediction (*RMSEP*), relative standard error of prediction (*RSEP*), absolute average deviation (*AAD*), and adequate precision (*AP*). One of the most important criteria to realize the model fitness quality is adj-*R*^2^, which is a modification of *R*^2^ to adjust the number of descriptive terms in a model. Hence, the adj-*R*^2^ unlike *R*^2^ is increased only when the new term improves the model more than would be expected by chance [37]. The high *R*^2^ (0.954–0.972) and adj-*R*^2^ (0.925–0.955) values confirmed the good fitness of response surface models for target responses. Low values of *CV* (0.13–3.37), *RMSEP* (0.00002–0.8547), *RSEP* (0.041–2.523), and *AAD* (0.214–76.21) show better reproducibility of data using the predictive models (Table 4). The *AP* value is a signal-to-noise ratio, which should be over 4.0. This factor compares the range of predicted values at fixed amounts to the mean prediction error [38]. In the current study, the *AP* values for response variables ranged from 20.80 to 27.77, indicating the sufficient coverage of experimental data with the constructed models.

#### 2.3.2. Physicochemical Quality Parameters

**Figure 3:**
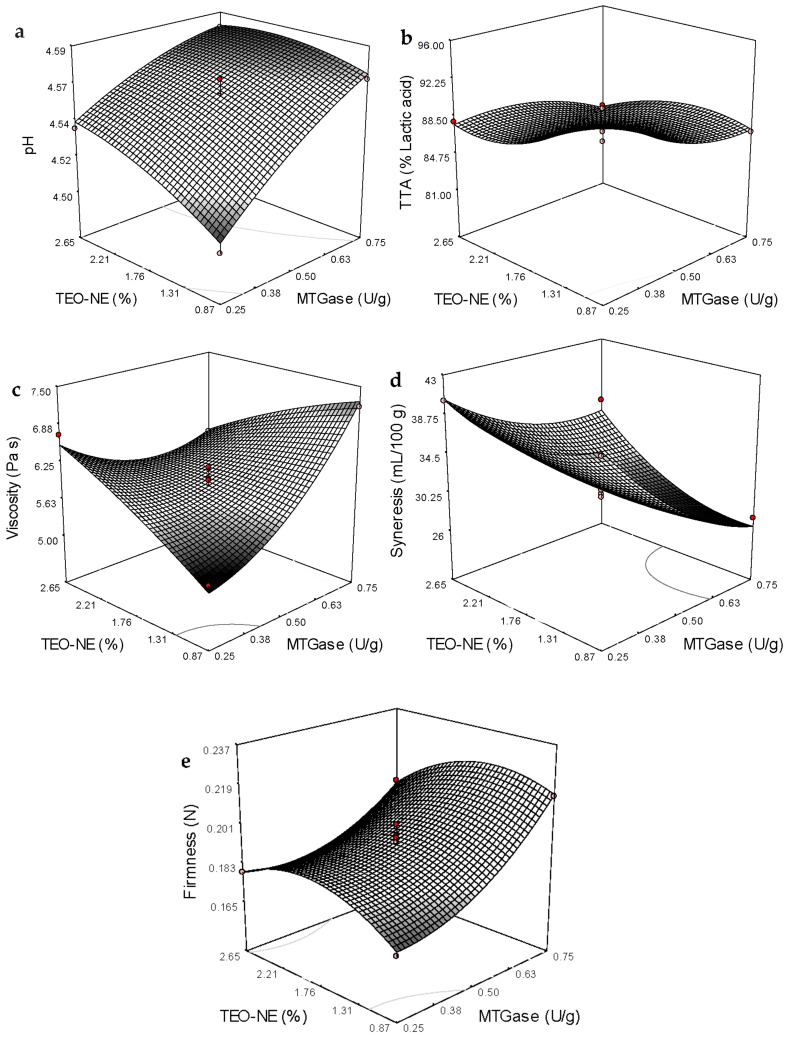
Three-dimensional surface plots showing the significant interaction effects on the variation of the pH (**a**), TTA (**b**), firmness (**c**), viscosity (**d**), and syneresis (**e**).

The ANOVA showed that the effect of TEO/SFO was stronger than tween-80 on the particle size and *PDI* results (*p* < 0.05). In general, an increase in the concentration A (Table 5) illustrates that the linear effect of MTGase concentration on all the response variables was highly significant (*p* < 0.0001). Except for the apparent viscosity, the addition level of TEO-NE to non-fat yogurt gels linearly affected the other response variables. The interaction effect of these two independent variables was also significant in all the response variables (Table 5). The quadratic effect of TEO-NE enrichment quantity was insignificant on the apparent viscosity. However, the quadratic term of MTGase concentration was significant in all the studied physicochemical quality responses (Table 4). Based on the sum of squares (Table 5) and coefficients in the fitted equations (Table 4), the linear term of MTGase concentration was the most significant (*p* < 0.05) effect on the pH, firmness, and syneresis. Moreover, the most significant effect on the total titratable acidity (TTA) value was revealed to be the quadratic effect of TEO-NE and the linear term of MTGase concentration. Likewise, the interaction between MTGase and TEO-NE had the maximum effect on the apparent viscosity of free-fat yogurt gels (Table 4 and Table 5). Figure 3a,b show that an increase in the incorporation level of MTGase and TEO-NE reduced the TTA and increased the pH value of yogurt gels. Adding the MTGase remarkably increased the viscosity and firmness, while a significant decrease in the syneresis was recorded at high MTGase levels (Figure 3c–e). The syneresis could be increased by increasing the incorporation level of TEO-NE (Figure 3e). Adversely, a decrease in the firmness was measured in yogurt gels containing a high TEO-NE percentage (Figure 3c).

The increased pH or reduced acidity in the presence of MTGase can hinder the growth of starter bacteria by limiting their accessibility to nitrogen sources such as amino acids through forming protein crosslinks. This complex protein matrix is able to retard lactic fermentation with a longer lag phase of starter bacteria, leading to a slower acidification with a lower production rate of organic acids, particularly LA [3]. Earlier, a rise in the pH value or a reduction in the acidification rate was reported in free-fat [39] and full-fat [40] set yogurts by increasing the MTGase concentration. An increase in the pH value with lowering acidification at high levels of TEO-NE addition may be attributed to the covering oil droplets in the NE by tween-80 layers, decreasing chemical reactions between the oil and water or air. Therefore, oils encapsulated in the NE system were more stable against hydrolysis and oxidation processes, resulting in less post-acidification of yogurt gels [41]. Similar results were reported for yogurts fortified with fish oil/γ-oryzanol nanoemulsions [41] and fish oil nanoliposomes [42].

**Figure 4:**
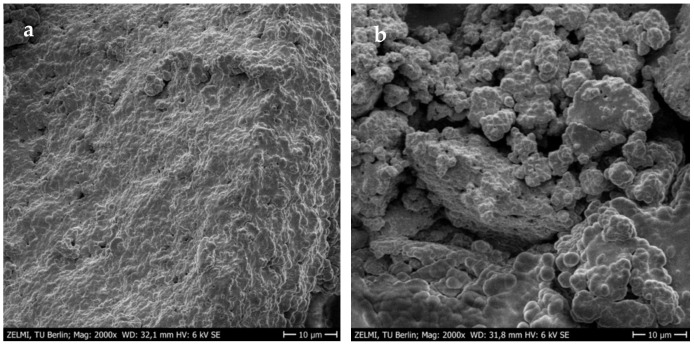
SEM images of 1.76% TEO-NE-containing yogurt gels treated with (**a**) and without 0.5 U/g MTGase (**b**).

The improved gel strength or firmness was more pronounced in yogurt networks treated with MTGase than in the control sample. It was not surprising because the cross-linking of milk proteins by MTGase through the formation of additional covalent (ε-(γ-glutamyl) lysine) bonds can strongly improve the yogurt gel strength [3]. A robust three-dimensional gel network between casein proteins can be formed via the inter- and intra-molecular crosslinks induced by MTGase. The formed network matrix with a high number of protein interactions remarkably improves the resistance against the deformation forces [8]. The scanning electron microscopy (SEM) images also confirmed that the incorporation of MTGase could provide a protein structure with more interconnected chains in order to improve the textural strength (Figure 4). It seems that alongside the covalent crosslinks, the hydrogen bonding formation between amino acid residues in milk proteins is responsible for the firmness improvement [3,43]. The high number of protein crosslinks or isopeptide linkages with β-casein induced by MTGase can guarantee structural stability with a more apparent viscosity. However, the incorporation level of MTGase is a determining factor in the increase of gel viscosity. It was previously proved that the maximum viscosity in different yogurts is a function of MTGase concentration. For example, the highest viscosity of MTGase-treated yogurt gels without any negative impact on the sensory properties was determined at 0.25 U/g in the mid-fat probiotic set [44], and 0.5 U/g in full/mid-fat set [45] and full-fat stirred [46] types. According to the individual optimization conducted in the present study, the use of MTGase at ~0.70 U/g resulted in the highest viscosity value in free-fat yogurt gels enriched with TEO-loaded NEs. Therefore, it is clear that the formation of a stable protein–MTGase complex matrix through increasing the entrapment of additional whey in the yogurt network can reduce the syneresis rate. This reduction would be increased at high MTGase concentrations by increasing the water holding capacity as a result of the reinforcement and stabilization of the three-dimensional yogurt gels [43]. Although the TEO-NE had no significant effect on the firmness and viscosity values, the syneresis was significantly reduced in the presence of high TEO-NE levels. The reason could be probably owing to the dilution of the yogurt gel with incorporating NEs with large quantities of water molecules [41].

#### 2.3.3. Optimization and Validation

**Figure 5:**
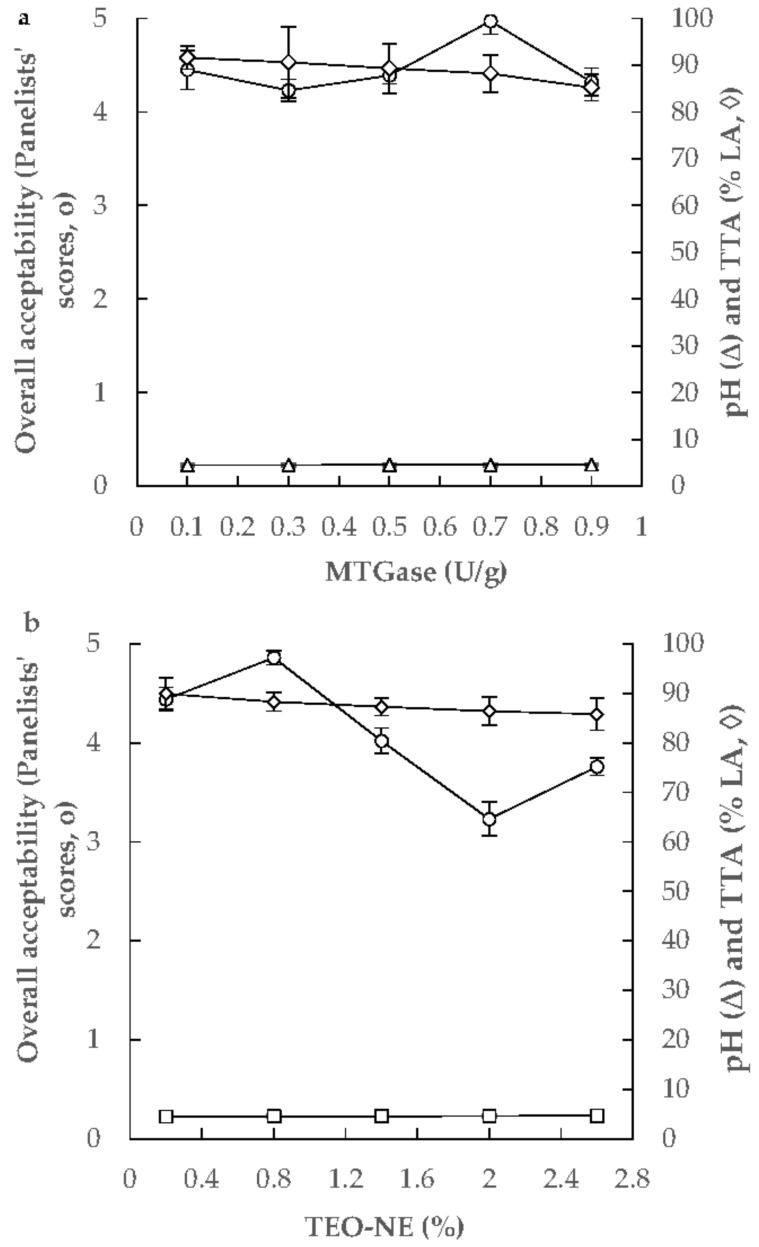
Changing the TTA, pH, and overall sensory acceptability of yogurt gels as a function of MTGase (0.1–0.9 U/g, with 0.75% TEO-NE) (**a**) and TEO-NE (0.2–2.6%, with 0.70 U/g MTGase) concentrations (**b**).

The numerical optimization procedure was performed to predict the optimum levels of MTGase and TEO-NE to achieve the desired pH and TTA, the minimum syneresis, and the maximum apparent viscosity and firmness of free-fat yogurt gels. Some preliminary studies were performed to achieve the desired levels of pH and TTA for yogurt gels. The pH, TTA, and overall acceptability of yogurt gels containing MTGase (0.1–0.9 U/g) and 0.75% TEO-NE showed that the use of 0.7 U/g MTGase could lead to the yogurt gel production with the maximum overall acceptability. The pH and TTA of this formulation were 4.570 and 88.2% LA, respectively (Figure 5a). In the next phase, the overall acceptability of yogurt gels formulated with 0.2–2.6% TEO-NE with 0.70 U/g MTGase was investigated. Results revealed that the yogurt gel composed of 0.80 TEO-NE presented the highest overall acceptability for panelists’ preferences. The pH and TTA of this formulation were 4.569 and 88.3% LA, respectively (Figure 5b). Therefore, the desired pH and TTA in the optimization study were considered to be 5.569 and 88.3% LA, respectively. The RSM package’s response optimizer revealed that the overall optimum region was at 0.87% (*w*/*w*) TEO-NE and 0.70 U/g MTGase. By substituting these values in the second-order polynomial models, the values of pH, TTA, syneresis, apparent viscosity, and firmness of free-fat yogurt gels were predicted to be 4.569, 88.3% LA, 27.03 mL/100 g, 6.93 Pa s, and 0.207 N, respectively. Under the optimum formulation, the corresponding experimental response values were 4.57 ± 0.02, 88.5 ± 0.9% LA, 29.17 ± 0.11 mL/100 g, 6.95 ± 0.29 Pa s, and 0.213 ± 0.017 N, respectively. As there was no significant difference between the actual and predicted data, it can be concluded that the fitted quadratic models were able to assess the physicochemical properties of these novel yogurt gels.

### 2.4. Sensory Attributes of Non-Fat Yogurt Gels

**Figure 6:**
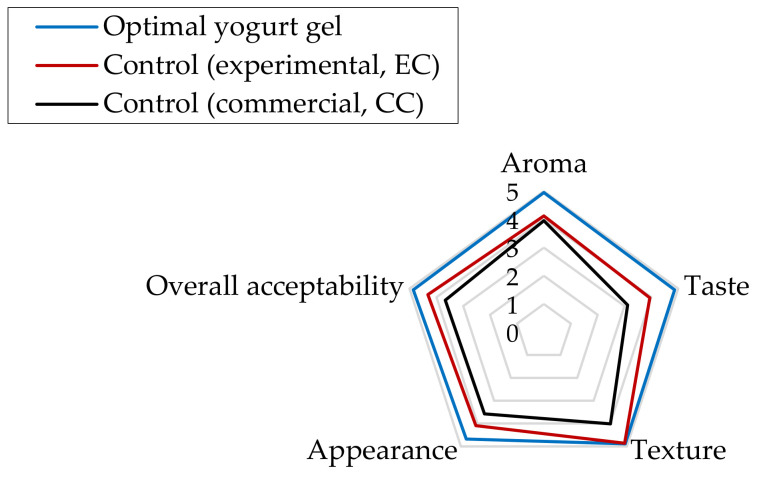
The organoleptic properties of the optimal yogurt gel compared two control samples (experimental and commercial).

Figure 6 shows that the optimal non-fat yogurt gel (0.87% TEO-NE and 0.70 U/g MTGase) compared to experimental control (EC, containing 0.70 U/g MTGase without TEO-NE) and free-fat commercial control (CC, containing modified starch E1422) samples obtained better organoleptic scores in aroma, taste, texture, appearance, and overall acceptability by sensory panelists. Furthermore, the EC was more preferred by panelists than the CC. However, there was no significant difference in the texture scores between the EC and the optimal sample (Figure 6). From the industrial viewpoint, the use of MTGase than modified starch is a better option not only for encapsulating bioactive compounds in yogurt gels but also for strengthening their textural characteristics. The presence of MTGase in the formulation of these yogurt gels could remarkably improve the texture scores by forming a complicated structural network with covalent solid crosslinks. Nevertheless, this protein matrix as an attractive carrier might well encapsulate TEO-NE in the optimal yogurt gel to release volatile compounds after digestion. Moreover, the chemical compounds present in TEO as carbon substrates were probably consumed by starter bacteria in lactic fermentation and presented a better taste for panelists.

### 2.5. Storage-Dependent Bioactivity of Optimal Yogurt Gels

The results of antimicrobial and antioxidant evaluations of the EC and optimum yogurt gels during cold storage for three weeks are given in Table 6. The inherent bioactivity of EC can be attributed to the presence of bioactive peptides with antioxidant activity in fermented milk products as a result of proteolysis of milk proteins [47]. Korhonen [48] pointed out that the released peptides during the fermentation step have some phenolic-side chains (such as tyrosine), which can potentially inhibit the formation of free radicals. In addition, new phenolic acids might be produced by utilizing phenolic compounds (e.g., ferulic and *p*-coumaric acids) during the lactic metabolism of microbes [49]. Moreover, there are some phenolic constituents as products from the secondary metabolism of plants in milk, which originated from ruminant feed [50,51]. In general, not only a lower count of molds and yeast was grown in the optimal formulation than the EC over the cold storage, but also a higher DPPH· inhibition rate was found for the optimal sample (*p* < 0.05). The better bioactivity of yogurts enriched with the optimal TEO-NE can be because of the presence of α-tocopherol and TEO in the oil phase of NEs. Vitamin E improves the antioxidant potential by quenching lipophilic free radicals via the phenolic hydrogen present in the chromanol ring. Furthermore, it seems that the use of MTGase could prevent vitamin E degradation during cold storage through oxidation induced by the presence of oxygen in yogurt and its synergist effect with light, heat, trace minerals, and hydroperoxides [52]. Dasgupta et al. proved that more antioxidant and antimicrobial activities for mustard oil/water NE loaded with vitamin E compared to the NE formulation without vitamin E [53]. The antimicrobial effect of vitamin E may be ascribed to its capability to cause perturbations in the integrity of bacterial cell membranes, enabling the penetration of antimicrobial agents of TEO [54]. The presence of TEO-NE is another reason for better antioxidant and antimicrobial potentials of the optimal formulation. The main cause of spoilage in yogurt products is yeast and mold contamination. Microbial growth prevention by TEO-NE can occur by disrupting the cell walls and membrane of microorganisms. Increasing the solubility of TEO during UAE can improve the release rate of bioactive volatile compounds to control the growth and activity of molds and yeasts. Furthermore, nanoemulsification can contribute to decreasing the interfacial tension and increasing the adsorption of these components on the microbial cells [31,34]. On the other hand, Chaleshtori et al., [23] also reported the high ability of TEO to inhibit linoleic acid oxidation. The antioxidant capacity of this EO can be related to the presence of oxygenated monoterpenes such as methyl chavicol and the high content of phenolics [23]. Moreover, many studies confirmed that the strong antiradical activity of other volatile compounds present in TEO quenches free radicals like DPPH· by donating electrons, such as linalool, limonene, geranial, eugenol, and α-pinene, β-pinene, etc. [55,56,57]. However, an increase in the growth of molds and yeasts and a reduction in the scavenging activity of DPPH radical (*SA*_DPPH·_) by prolonging the cold storage time were observed (*p* < 0.05, Table 6). The decrease in the antioxidant and antimicrobial activities of yogurts during cold storage may be associated with the degradation of phenolic compounds and increased interactions between milk proteins and polyphenols [58]. It can be hypothesized that the presence of MTGase in the structural network can retard the degradation rate of TEO, vitamin E, and phenolics, leading to the improved bioactivity of the optimum yogurt formulation.

## 3. Conclusions

In this work, innovative MTGase-induced non-fat yogurt gels were developed and enriched with TEO-loaded NEs. Methyl chavicol was the most dominant bioactive component of TEO extracted by the hydrodistillation process. The UAE process resulted in the fabrication of physiochemically stable NEs with 0.5% tween-80 and 1:2 TEO/SFO based on the droplet size, *DGR*, and PV values during the 30-day storage. The RSM-CCRD using second-order polynomial models could successfully predict the optimal formulation of free-fat yogurt gels fortified with TEO-NEs with the maximum firmness and apparent viscosity, the desirable pH and TTA, as well as the minimum syneresis rate. The SEM images proved that the textural improvement of yogurt gels was possible by inducing MTGase crosslinking. The sensory evaluation of the optimal gels compared to two commercial and experimental control samples showed that the presence of MTGase emended the polymeric texture and structure to encapsulate TEO-NE for the targeted release of taste and aroma properties for panelists. Bioactive compounds present in this optimal sample could well control the growth and activity of molds and yeasts and also the oxidation chain reaction during cold storage. Further studies should be performed to assess the lipolysis and bioaccessibility rate of TEO-NE during the in vitro digestion conduction. Another evaluation should be the effect of MTGase and TEO-NE on the survivability of starter bacteria in the classical elastic gel network or the viability of probiotic bacteria in probiotic/synbiotic yogurt gels in the future. It is also recommended to produce nano-scale powders of TEO-NE using different technologies to fortify similar yogurt gels for comparison with the outcomes of the present study.

## 4. Materials and Methods

### 4.1. Milk, Yogurt Starter, Culture Medium, and Chemicals

Skim milk (~0.1% fat) was purchased from a local supermarket in Berlin (Germany). The free-fat CC yogurt containing cow milk, free-fat milk concentrate, protein powder of milk, yogurt starter, modified starch (E1422), and refined edible salt was purchased from a retail market. The freeze-dried yogurt starter culture of YO-FAST-88 (*Str. thermophilus* and *Lb. delbrueckii* subsp. *bulgaricus*) was purchased from Chr. Hansen Co. (Hoersholm, Denmark). Yeast extract glucose chloramphenicol (YGC) agar medium was purchased from Merck Chemical Co. (Darmstadt, Germany). MTGase (Activa^®^ YG, 1000 U/g) was provided by Ajinomoto Co., Inc. (Hamburg, Germany). Ascorbic acid, tween-80, isooctane, isopropanol, chloroform, methanol, ammonium thiocyanate (NH_4_SCN), ferrous chloride (FeCl_3_), potassium sorbate, sodium acetate, sodium hydroxide (NaOH), anhydrous sodium sulfate, vitamin E (α-tocopherol), phenolphthalein, and 1,1-diphenyl-2-picrylhydrazyl (DPPH) were purchased from Sigma-Aldrich Chemical Co. (Darmstadt, Germany).

### 4.2. Plant Collection and Hydrodistillation Process of TEO Extraction

Fresh tarragon (*A. dracunculus* L.) was purchased from a local grocery (Berlin, Germany). Leaves were separated, sun-dried, and re-dried in a vacuum oven at 60 ± 1 °C for one day to attain a constant weight. After crushing with a mortar and passing through a sieve with a 2 mm mesh, tarragon powders (~300 g) and water (450 mL) were mixed in a round bottom flask and connected to a Clevenger-type apparatus to perform the hydrodistillation. This process was completed after 4 h from the boiling start. The collected TEO was dried over anhydrous sodium sulfate and stored in a refrigerator at 4 °C before analyzing the chemical composition and nano-emulsifying.

### 4.3. Gas Chromatography–Mass Spectrometry Analysis

A gas chromatography (GC, Agilent 6890, Santa Clara, CA, USA) equipped with a Chrome-pack BPX5 capillary column (30 m × 250 μm × 0.25 μm; stationary phase, 5% phenyl 95% methyl polysiloxane) and coupled with an ionization mass detector (MS, Agilent 5973N, Palo Alto, CA, USA) were used to analyze chemical compounds of TEO. Helium gas was used as a carrier gas at a flow rate of 1.0 mL/min with a split ratio injection of 1:30. Moreover, the temperature of injection was 250 °C. The oven temperature program was as follows: initially set at 40 °C (isothermal for 1 min), gradually increased to 250 °C with a rate of 3 °C/min, and finally, isothermally kept at this temperature for 10 min. The MS procedure was operated through ionization energy of 70 eV. The scan interval and range were set at 0.5 s and 50–550 *m*/*z*, respectively [31]. Each individual quantified constituent was identified based on the following criteria: calculated Kovats retention indices compared to Adams libraries and the comparison of mass fragmentation pattern in the NIST mass spectral library [59,60].

### 4.4. TEO-Nanoemulsion Preparation

The oil phase (10% *w*/*w*) prepared by the mixture of TEO and SFO (2:1, 1:1, and 1:2 *w*/*w*) and α-tocopherol (α-tocopherol/oil phase 1:5) were dispersed in the aqueous phase containing tween-80 (0.2, 0.5, and 0.8% *w*/*w*) and potassium sorbate (0.1% *w*/*w*) in an aqueous buffer solution of sodium acetate (10 mM, pH 4.0). The initial homogenization was performed by a high-speed blender (Ultra-Turrax, IKA T25 Digital, Staufen, Germany) at 12,500 rpm for 2.5 min and the emulsion premixes with coarse droplets were then sonicated to produce TEO-NEs with fine droplets using a 20 kHz ultrasonic apparatus (UP200S, Hielscher Ultrasonics GmbH, Teltow, Germany) equipped with a 13-mm-diameter titanium sonotrode probe at a total nominal output power of 175 W for 20 min [61]. The temperature difference from micro-emulsions to NEs during emulsification was maintained below 20 °C by holding the vessel in a refrigerated water bath. The ideal operating conditions in both homogenization steps were determined to achieve the maximum physicochemical stability based on the preliminary tests.

### 4.5. Particle Size Analysis of Nanoemulsions

The *D*_43_ of TEO-NE droplets stored in 30 mL Nalgene tubes after diluting with deionized water (1:100) was measured using the Mastersizer 2000S particle size analyzer (Malvern Instruments Ltd., Worcestershire, UK). This parameter was estimated by the following formula (Equation (1)):

(1) $$D_{43}=\frac{\sum n_{i}d_{i}^{4}}{\sum n_{i}d_{i}^{3}}$$

where *n_i_* is the number of droplets of radius *d_i_*.

Further, the distribution width of droplet size (Span-value or *PDI*) was calculated as follows (Equation (2)):

(2) $$PDI=\frac{\left(d\left(\nu ,\;\;90\right)-d\left(\nu ,\;\;10\right)\right)}{\left(\nu ,\;\;50\right)}$$

where *d*(*υ*, 10), *d*(*υ*, 50), and *d*(*υ*, 90) are diameters at 10, 50, and 90% cumulative volume, respectively. In other words, [*d*(*υ*, 90)–*d*(*υ*, 10)] and *d*(*υ*, 50) are the range and median diameter amounts, respectively [27].

### 4.6. Physical Instability Measurement

Increasing the size of NE droplets during 30-day storage in 30 mL Nalgene tubes under ambient temperature was considered as a criterion of the physical instability. The *DGR* was calculated as follows (Equation (3)) [29]:

(3) $$DGR=\frac{\left(D_{43}^{d30}-D_{43}^{d0}\right)}{D_{43}^{d0}}$$

where $D_{43}^{d0}$ and $D_{43}^{d30}$ are the volume-weighted mean diameter of fresh and 30-day-stored NEs, respectively.

### 4.7. Chemical Instability Determination

The procedure of Osborn and Akoh [62] with minor modifications was used to extract the oil from TEO-NEs. For this work, 15 mL of NE aliquots stored in 30 mL Nalgene tubes at room temperature for the oxidation progression were transferred into capped test tubes and placed in a water bath at a constant temperature of 50 ± 1 °C. A mixture of isooctane/isopropanol (3:2, *v*/*v*) was added to NE aliquots, vortexed for 30 s, and centrifuged (134× *g*, 5 min, 25 °C) to extract the oil phase. The oil samples were obtained after the supernatant removal and the solvent evaporation under nitrogen gas. The International Dairy Foundation method as described by Gharibzahedi et al. [29,33] was applied to determine the oil oxidation rate over storage for 30 days at ambient temperature based on the PV measurement. In brief, about 0.25 g of the extracted oil into a glass test tube was weighed and dissolved in chloroform/methanol (9.7 mL; 4:1, *v*/*v*). After adding a drop of each solution of NH_4_SCN (30%, *w*/*v*) and FeCl_3_ (0.35%, *w*/*v*) and holding for 5 min, the mixture absorbance was measured using a UV-visible spectrophotometer (Jenway 7315, Staffordshire, UK) at 500 nm. A standard calibration curve with FeCl_3_ solutions containing 5–20 μg of ferric ions was drawn to express results as meq O_2_/kg oil.

### 4.8. Preparation of Free-Fat Yogurt Gels

The best TEO-NE formulation at different levels (0.5–3.02% *w*/*w*, Table 3) was mixed with skim milk enriched with 2% skimmed milk and whey powders (1:1 *w*/*w*) using an IKA T25 Ultra-Turrax blender at 7500 rpm for 7.5 min. The milky mixture was then heated to pasteurize at 85 °C for 5 min, immediately cooled to 42 ± 1 °C to add MTGase (0.15–0.85 U/g protein, Table 1), and incubated before fermentation for 2 h at the same temperature. The enzyme activity was stopped by heating for 1 min at 80 °C. In the next step, the commercial starter was inoculated and incubated at 43 ± 1 °C until pH 4.3–4.4 was reached. The developed non-fat set-yogurts were then stored at 4 °C for three weeks. Each yogurt formulation was produced in duplicate (Table 3).

### 4.9. Total Titratable Acidity and pH Assessment

A Voltcraft pH-100 ATC pH-meter was used to determine the pH of yogurt samples, while it was daily calibrated with pH 4.0 and 7.0 buffer solutions. The TTA was measured by titrating 9 g of yogurt with 0.1 N NaOH using phenolphthalein as an indicator and expressed as a percentage of LA [63].

### 4.10. Firmness and Viscosity Measurements

The yogurt texture was evaluated by measuring the firmness using a Zwick texture analyzer (Roller Company, Ulm, Germany) equipped with a 50 kg compression load cell. An integrator was used to carry out the puncture test using a stainless-steel 3-mm-diameter probe and a test velocity of 1 mm/s. The firmness value was defined as the highest peak force and determined from the penetration curve. A steady-stress rheometer (Brookfield DV-II+, Stoughton, MA, USA) equipped with a spindle (no. 4) was also used to measure the apparent viscosity of yogurt samples (~75 mL) at ambient temperature [1].

### 4.11. Syneresis Evaluation

One hundred grams of yogurt samples were placed on a Whatman filter paper setting on top of a funnel. The serum volume collected in a graduated cylinder after the drainage at 7 °C for 2 h was considered as syneresis index [64].

### 4.12. Experimental Design and Modeling Studies

An RSM-CCRD using the software package of Design-Expert (trial version 7.1.3, Stat-Ease Inc., Minneapolis, MN, USA) was applied for experimental design, data analysis, mathematical modeling, and numeric optimization. Two independent parameters of MTGase (X_1_, 0.15–0.85 U/g) and TEO-NE (X_2_, 0.5–3.02% *w*/*w*) with five levels chosen for each variable were assessed. Response variables included pH (Y_1_), TTA (Y_2_), firmness (Y_3_), viscosity (Y_4_), and syneresis (Y_5_) of free-fat yogurts. Based on RSM-CCRD, 14 trials each at five coded levels (i.e., −1.41, −1, 0, 1, and 1.41) with six center points were designed (Table 2). Each response function (Y) related to the coded variables (X_i_, i = 1, 2) was fitted by a second-order polynomial equation (Equation (4)):

(4) $$Y=\beta_{0}+\beta_{1}X_{1}+\beta_{2}X_{2}+\beta_{12}X_{1}X_{2}+\beta_{11}X_{1}^{2}+\beta_{22}X_{2}^{2}$$

The coefficients present in this formula were represented by β_0_ (constant term), β_1_ and β_2_ (linear effects), β_11_ and β_22_ (quadratic effects), and β_12_ (interaction effects) [65].

The fitness quality of quadratic models was evaluated according to the *CV*, *R*^2^, adj-*R*^2^, *PRESS*, *RMSEP*, *RSEP*, *ADD*, and *AP* amounts (Equations (5)–(11)):

(5) $$R^{2}=1-\frac{SS_{Residual}}{\left(SS_{Model}+SS_{Residual}\right)}$$

(6) R2=1−SSResidualDFResidualSSModel+SSResidualDFModel+DFResidual

(7) $$RMSEP=\sqrt{\frac{\sum_{i=1}^{N}{\left(y_{pre,i}-y_{exp,i}\right)}^{2}}{N}}$$

(8) $$RSEP=\left(\sqrt{\frac{\sum_{i=1}^{N}{\left(y_{pre,i}-y_{exp,i}\right)}^{2}}{\sum_{i=1}^{N}{\left(y_{exp,i}\right)}^{2}}}\right)\times 100$$

(9) $$ADD=\left(\frac{\sum_{i=1}^{N}\left(\left|y_{exp,i}-y_{pre,i}\right|\right)}{N}\right)\times 100$$

(10) AP=maxy¯−miny¯V¯y¯

where

(11) $$\bar{V}\left(\bar{y}\right)=\frac{1}{N}\sum_{i=1}^{N}V\left(\bar{y}\right)=\frac{n\sigma^{2}}{N}$$

In the above equations, *SS* and *DF* are the sum of squares and the degrees of freedom, respectively. *y_exp,i_* and *y_pre,i_* are the experimental and predicted responses, respectively. *n* and *N* are the numbers of model factors and experiments, respectively. $\bar{y}$ and *σ*^2^ are the predicted value and the residual mean square from the ANOVA table [37].

For achieving the final reduced models, non-significant (*p* > 0.05) terms were removed from the initial models. Further, five extra confirmation tests were performed to verify the validity of the data predicted under the theoretically optimal formulation by RSM-CCRD. The validity of the fitted models was confirmed by comparing the actual and predicted data according to Student’s t-test using SPSS V.21 (SPSS Inc., Chicago, IL, USA) software at a significant level of 5%.

### 4.13. Microscopy Analysis

The microstructure of freeze-dried yogurt samples (ALPHA 2–4LD Plus freeze-dryer, Christ, Osterode am Harz, Germany) was subjected to metallization (sputtering, Balzers Union, model FL 9496) with a thin layer of a conductive gold coating for 30 s to amplify the secondary electron signal. They were then visualized using a field emission-SEM (FE-SEM, Zeiss Gemini DSM 982, LEO, Oberkochen, Germany) at a 10 μm scale bar under 2000× magnification and an accelerating voltage of 6 kV.

### 4.14. Sensory Attributes Evaluation

The sensory properties of samples (optimum, EC, and CC) in terms of aroma, taste, texture, color/appearance, and overall acceptability were assessed by 17 trained panelists, who were familiar with the basic qualities of yogurt products. These people were selected among 29 participants with the ability to replicate their sensory results. The final panelists were 9 females and 8 males within an age range between 21 to 49 years and evaluated each sample twice (~20 g) in a random order in sensory booths with standard lighting. Panelists were asked to drink water to rinse their mouths between samples while some unsalted crackers were available. They filled out the data in a questionnaire with a hedonic 5-point scale, in which 5 corresponded to “most liked” and 1 to “most disliked” [66].

### 4.15. Antifungal Activity Determination

The count of molds and yeasts of yogurt samples during 21-day cold storage was evaluated based on the method described by El Omari et al. [67]. After the culture of samples in plates with YGC medium, they were aerobically incubated in a refrigerated incubator (25 ± 1 °C for 3–5 days), and then the colonies were counted. A yogurt sample prepared without TEO-NE was used as the control sample.

### 4.16. Antioxidant Activity Determination

Fresh and stored (7, 14, and 21 days) yogurt samples were initially centrifuged at 537× *g* and 4 °C for a half hour, and the supernatant was filtered through a 0.45 μm-membrane filter to determine the inhibition rate of DPPH radical. 40 μL of each supernatant (commercial and optimal formulations) was added to 2.9 mL of DPPH solution (0.1 mM), incubated in the dark for 30 min, and the mixture absorbance was read at 517 nm using a UV−visible spectrophotometer. The positive and negative control samples were ascorbic acid (0.1 mg/mL) and the yogurt sample prepared without TEO-NE. The *SA*_DPPH·_ rate of yogurt samples was calculated by the following equation (Equation (12)) [68]:

(12) $$SA_{\mathrm{DPPH}\cdot }=\frac{A_{c}-A_{s}}{A_{s}}\times 100$$

where *A_c_* and *A_s_* are the absorbance of negative-control and sample, respectively.

### 4.17. Statistical Analysis

All the experiments related to NEs’ physiochemical characteristics as well as antioxidant, antimicrobial, and sensory properties of yogurt samples during the cold storage were performed in triplicate and the results were represented as a mean ± SD. The results were subjected to ANOVA, and Duncan’s multiple range was conducted to compare the means using SPSS v.22 (SPSS Inc., Chicago, IL, USA) software. A *p*-value ≤ 0.05 was statistically significant

## Figures and Tables

**Table 1:** Major chemical constituents of TEO analyzed by GC-MS.

Constituent	Structure	RT (min)-KI ^1^	Quantity (%)
α-Pinene	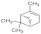	11.21–927	0.67
β-Pinene	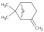	13.30–969	0.19
β-Myrcene	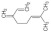	14.19–986	0.15
Limonene	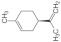	15.99–1025	0.91
z-β-Ocimene	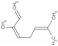	16.72–1037	4.79
Trans-β-ocimene	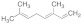	17.23–1047	4.02
Estragole (Methyl chavicol)	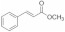	25.25–1226	85.66
Geranial	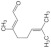	28.05–1270	0.26
Eugenol	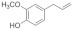	31.81–1353	0.15
(E)-Isosafrole	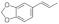	32.88–1377	0.45
4-Methoxy cinnamaldehyde	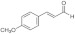	40.43–1561	0.16

**^1^** RT: Retention time, KI: Kovats indices.

**Table 2:** Storage-dependent droplet growth ratio (*DGR*) and peroxide value (PV) changes of TEO-NEs at different TEO/SFO ratios and tween-80 (T-80) levels for 30 days at room temperature.

TEO/SFO Ratio	Storage (Day)	Physical Instability (*DGR*) ^1^			Chemical Instability (PV, meq O_2_/kg oil) ^1^		
		0.2% T-80	0.5% T-80	0.8% T-80	0.2% T-80	0.5% T-80	0.8% T-80
2:1	0	297.8 ± 3.4 ^fA^	254.8 ± 2.1 ^dB^	192.4 ± 3.7 ^eC^	0.909 ± 0.008 ^dA^	0.654 ± 0.003 ^dB^	0.623 ± 0.006 ^fB^
	7	327.8 ± 5.9 ^eA^	267.0 ± 11.0 ^dB^	242.7 ± 5.5 ^dB^	0.919 ± 0.006 ^dA^	0.701 ± 0.001 ^cB^	0.672 ± 0.009 ^eB^
	15	387.9 ± 11.1 ^dA^	303.7 ± 10.8 ^cB^	278.0 ± 9.7 ^cB^	0.978 ± 0.012 ^cA^	0.770 ± 0.002 ^bB^	0.711 ± 0.002 ^dC^
	23	471.3 ± 12.4 ^cA^	400.8 ± 8.9 ^bB^	334.0 ± 7.5 ^bC^	1.043 ± 0.004 ^bA^	0.824 ± 0.010 ^aB^	0.805 ± 0.000 ^bB^
	30	602.3 ± 7.1 ^aA^	478.9 ± 6.5 ^aB^	341.6 ± 8.7 ^bC^	1.121 ± 0.015 ^aA^	0.842 ± 0.004 ^aB^	0.851 ± 0.007 ^aB^
1:1	0	232.1 ± 4.3 ^gA^	132.8 ± 5.2 ^gC^	189.9 ± 2.7 ^eB^	0.842 ± 0.008 ^eA^	0.654 ± 0.003 ^dB^	0.623 ± 0.006 ^fB^
	7	291.5 ± 6.3 ^fA^	175.1 ± 10.9 ^fB^	200.3 ± 6.7 ^eB^	0.898 ± 0.012 ^dA^	0.607 ± 0.007 ^eB^	0.641 ± 0.013 ^efB^
	15	327.8 ± 11.1 ^eA^	202.6 ± 9.1 ^eC^	275.7 ± 8.8 ^cB^	0.907 ± 0.007 ^dA^	0.677 ± 0.000 ^cdC^	0.721 ± 0.007 ^dB^
	23	416.5 ± 7.7 ^dA^	298.9 ± 6.5 ^cC^	367.9 ± 7.3 ^aB^	1.023 ± 0.018 ^bA^	0.754 ± 0.003 ^bB^	0.764 ± 0.008 ^cB^
	30	518.0 ± 9.0 ^bA^	314.7 ± 3.0 ^cC^	379.8 ± 5.9 ^aB^	1.098 ± 0.029 ^aA^	0.819 ± 0.010 ^aB^	0.829 ± 0.004 ^abB^
1:2	0	149.9 ± 6.2 ^iA^	78.9 ± 7.9 ^iC^	116.3 ± 8.7 ^gB^	0.761 ± 0.002 ^fA^	0.656 ± 0.002 ^dB^	0.633 ± 0.011 ^fB^
	7	161.87 ± 4.3 ^iA^	88.1 ± 8.8 ^iC^	121.6 ± 9.8 ^gB^	0.779 ± 0.002 ^fA^	0.680 ± 0.006 ^cdB^	0.641 ± 0.009 ^efB^
	15	191.1 ± 4.4 ^hA^	103.3 ± 2.9 ^hC^	156.0 ± 5.9 ^fB^	0.856 ± 0.009 ^eA^	0.698 ± 0.007 ^cB^	0.680 ± 0.001 ^eB^
	23	229.5 ± 9.2 ^gA^	142.1 ± 7.7 ^gC^	190.4 ± 6.7 ^eB^	0.899 ± 0.003 ^dA^	0.712 ± 0.005 ^cB^	0.702 ± 0.000 ^dB^
	30	293.8 ± 10.5 ^fA^	189.9 ± 4.8 ^efC^	231.5 ± 9.8 ^dB^	0.943 ± 0.010 ^cdA^	0.763 ± 0.001 ^bB^	0.754 ± 0.004 ^cB^

**^1^** Means with different superscript letters in each row (A–C) and column (a–i) indicate the significant statistical difference (*p* < 0.05).

**Table 3:** Experimental design matrix for CCRD-RSM and actual responses.

Run No.	Point Type	Independent Variables		Response Variables				
		MTGase (U/g)	TEO-NE (%)	pH	TTA (% LA)	Firmness (N)	Viscosity (Pa s)	Syneresis (mL/100 g)
1	Factorial	0.25	0.87	4.50 ± 0.01	94.0 ± 1.0	0.170 ± 0.007	5.20 ± 0.12	39.76 ± 0.10
2	Factorial	0.75	0.87	4.57 ± 0.00	87.2 ± 2.9	0.214 ± 0.003	7.17 ± 0.08	27.41 ± 0.09
3	Factorial	0.25	2.65	4.54 ± 0.01	88.0 ± 2.1	0.179 ± 0.005	6.71 ± 0.04	40.26 ± 0.08
4	Factorial	0.75	2.65	4.58 ± 0.02	85.1 ± 1.1	0.201 ± 0.000	6.05 ± 0.07	35.76 ± 0.06
5	Axial	0.15	1.76	4.52 ± 0.00	88.5 ± 1.0	0.193 ± 0.006	5.96 ± 0.09	42.79 ± 0.06
6	Axial	0.85	1.76	4.59 ± 0.01	80.9 ± 3.9	0.237 ± 0.005	7.45 ± 0.11	27.03 ± 0.06
7	Axial	0.50	0.50	4.54 ± 0.02	96.0 ± 1.2	0.173 ± 0.004	5.65 ± 0.12	32.45 ± 0.09
8	Axial	0.50	3.02	4.57 ± 0.02	89.1 ± 2.3	0.165 ± 0.004	5.78 ± 0.05	37.90 ± 0.11
9	Center	0.50	1.76	4.56 ± 0.00	86.0 ± 2.1	0.187 ± 0.002	5.98 ± 0.04	31.78 ± 0.16
10	Center	0.50	1.76	4.56 ± 0.01	86.9 ± 0.9	0.196 ± 0.001	6.17 ± 0.04	30.09 ± 0.09
11	Center	0.50	1.76	4.56 ± 0.02	87.1 ± 0.5	0.201 ± 0.002	5.83 ± 0.07	29.76 ± 0.07
12	Center	0.50	1.76	4.57 ± 0.02	89.0 ± 1.9	0.193 ± 0.003	5.95 ± 0.09	31.59 ± 0.03
13	Center	0.50	1.76	4.56 ± 0.01	87.9 ± 1.7	0.195 ± 0.002	5.95 ± 0.13	32.31 ± 0.03
14	Center	0.50	1.76	4.57 ± 0.00	86.9 ± 3.2	0.191 ± 0.002	5.70 ± 0.08	30.56 ± 0.06

**Table 4:** Final reduced second-order polynomial models and their fitness quality parameters for response variables.

Response Variable	2nd-Order Polynomial Model	Fitness Factor						
		*R* ^2^	adj-*R*^2^	*CV*	*RMSEP*	*RSEP*	*A* *AD*	*AP*
pH	Y_1_ = 4.56 + 0.026X_1_ + 0.012X_2_ – 0.007X_1_X_2_ – 0.006X_1_^2^–0.006X_2_^2^	0.964	0.942	0.13	0.0059	0.131	0.357	21.66
TTA	Y_2_ = 87.33 – 2.49X_1_ – 2.24X_2_ + 1.0X_1_X_2_ – 1.42X_1_^2^ + 2.58X_2_^2^	0.965	0.944	0.97	0.6429	0.730	45.14	26.37
Firmness	Y_3_ = 19 + 0.016X_1_ – 0.001X_2_ – 0.005X_1_X_2_ + 0.01X_1_^2^ – 0.013X_2_^2^	0.972	0.955	2.04	0.00002	2.390	0.214	27.77
Viscosity	Y_4_ = 5.93 + 0.43X_1_ – 0.66X_1_X_2_ + 0.41X_1_^2^	0.954	0.925	2.71	0.1249	0.041	10.07	20.80
Syneresis	Y_5_ = 31.02 – 4.89X_1_ + 2.07X_2_ + 1.96X_1_X_2_ + 2.14X_1_^2^ + 2.27X_2_^2^	0.967	0.947	3.37	0.8547	2.523	76.21	21.22

**Table 5:** ANOVA table for the independent and response variables ^1^.

Source	*DF*	pH		TTA (% LA)		Firmness (N)		Viscosity (Pa s)		Syneresis (mL/100 g)	
		*SS*	*p*-Value	*SS*	*p*-Value	*SS*	*p*-Value	*SS*	*p*-Value	*SS*	*p*-Value
Model	5	0.007	<0.0001	162.2	<0.0001	0.0043	<0.0001	4.55	<0.0001	307.75	<0.0001
X_1_	1	0.005	<0.0001	49.50	<0.0001	0.002	<0.0001	1.46	<0.0001	191.47	<0.0001
X_2_	1	0.001	0.0005	40.05	<0.0001	0.00002	ns	0.04	ns	34.27	0.0008
X_1_X_2_	1	0.0002	0.0321	4.0	0.0466	0.0001	0.0231	1.73	<0.0001	15.41	0.0084
X_1_^2^	1	0.0002	0.0220	14.82	0.0019	0.0007	<0.0001	1.22	0.0002	33.70	0.0009
X_2_^2^	1	0.0002	0.0220	49.28	<0.0001	0.011	<0.0001	0.06	ns	38.01	0.0006
Residual	7	0.0002		5.79		0.0000		0.22		10.23	
LoF	3	0.0001	0.2843 ^ns^	0.45	0.9309 ^ns^	0.0000	0.9223 ^ns^	0.09	0.3759 ^ns^	5.00	0.3023 ^ns^
Pure error	4	0.0001		5.33		0.0001		0.12		5.23	
Cor Total	13	0.0075		168		0.0046		4.77		317.98	

^1^ DF: Degree of freedom, SS: Sum of squares, LoF: Lack-of-fit, LA: Lactic acid, ^ns^: Non-significant, X_1_ (MTGase concentration), X_2_ (addition level of TEO-NE).

**Table 6:** Cold storage-dependent antifungal and antioxidant activities of optimal yogurt gels supplemented with TEO-NEs compared to the optimal sample.

Storage (Day)	Mold and Yeast Count (CFU/g) ^1^		Antioxidant Activity (*SA*_DPPH_, %) ^1^	
	Optimum	Control	Optimum	Control
0	0 ± 0.00 ^dA^	0 ± 0.00 ^dA^	56.78 ± 2.65 ^aA^	36.70 ± 0.87 ^aB^
7	0.31 ± 0.04×10^2 cB^	3.75 ± 0.32×10^2 cA^	53.43 ± 1.65 ^aA^	30.11 ± 0.40 ^bB^
15	0.92 ± 0.13×10^2 bB^	7.11 ± 1.76×10^2 bA^	42.87 ± 3.42 ^bA^	24.67 ± 1.46 ^cB^
21	1.21 ± 0.24×10^2 aB^	12.05 ± 0.78×10^2 aA^	39.56 ± 1.24 ^bA^	11.54 ± 1.59 ^dB^

^1^ Means with different superscript letters in each row (A–B) and column (a–d) indicate the significant statistical difference (*p* < 0.05).

## Data Availability

Not applicable.
